# Description of a priest in a medieval Japanese essay, "Tsurezuregusa": is this the first report of frontotemporal dementia?

**DOI:** 10.1590/1980-5764-DN-2025-0383

**Published:** 2026-04-20

**Authors:** Yuichiro Inatomi, Minoru Matsuda

**Affiliations:** 1 Saiseikai Kumamoto Hospital, Department of Neurology, Kumamoto, Japan.; 2 Izumi-no-mori Clinic, Sendai, Japan.

**Keywords:** Frontotemporal Dementia, Frontotemporal Lobar Degeneration, Dementia, Cognitive Reserve, Demência Frontotemporal, Degeneração Lobar Frontotemporal, Demência, Reserva Cognitiva

## Abstract

In Tsurezuregusa, a medieval Japanese essay written by Kenkō, a priest with the characteristics of behavioral variant frontotemporal dementia (bvFTD), has been described. He had characteristics such as early behavioral disinhibition; loss of sympathy and empathy; perseverative, stereotyped, and compulsive/ritualistic behavior; and hyperorality and dietary changes. Based on the diagnostic criteria for bvFTD, it is possible that he had probable bvFTD. According to the essay, despite his abnormal behavior, the priest had adapted to his surroundings, including the temple where he worked. His successful adaptation was likely due to the tolerance of people around him and/or the education he had acquired before the onset of the disease, which may have functioned as a "cognitive reserve."

## RESUMO.

Em Tsurezuregusa, foi descrito um ensaio medieval japonês escrito por Kenkō, um sacerdote com características de demência frontotemporal variante comportamental (DFTc). Kenkō apresentava características como desinibição comportamental precoce, perda de simpatia e empatia, comportamento perseverativo, estereotipado e compulsivo/ritualístico, hiperoralidade e alterações alimentares. Com base nos critérios diagnósticos para DFTc, é possível que ele tivesse DFTc provável. De acordo com o ensaio, apesar de seu comportamento anormal, o sacerdote havia se adaptado ao ambiente, incluindo o templo onde trabalhava. Sua adaptação bem-sucedida provavelmente se deveu à tolerância das pessoas ao seu redor e/ou à educação que havia adquirido antes do início da doença, que pode ter funcionado como uma reserva cognitiva.

## INTRODUCTION

 Frontotemporal dementia was first described by Pick, a Czech neurologist, in 1892^1,2^. "Tsurezuregusa" is an essay written by Kenkō (Urabe Kenkō, Yoshida Kenkō, 1280–1353), a Japanese Buddhist priest^3,4^. He wrote an essay between 1330 and 1332. The work is widely considered a gem of medieval Japanese literature and one of three representative essays, along with the "Makura no Sōshi": Pillow Book and "Hōjōki." "Tsurezuregusa" comprises a preface and 243 chapters, varying in length from a single line to a few pages. 

 In this essay, Kenkō wrote about Buddhist truths and themes such as death and impermanence that prevailed in the work. On the other hand, he also wrote some accounts of humorous incidents. 

 Chapter 60 of this essay describes a priest who may have had behavioral variant frontotemporal dementia (bvFTD), which we report here. This priest named "Jōshin" is believed to have been one of the scholars at Shinjō-in Temple, where the head priest was the son of the Kamakura shogunate regent. Kenkō did not meet the priest in person and was believed to have learned his words and actions through his acquaintances^4^. 

## TSUREZUREGUSA CHAPTER 60 TRANSLATED BY THE AUTHORS FROM CLASSICAL JAPANESE TO MODERN ENGLISH)

 At Shinjō-in Temple, a sub-temple of Ninna-ji Temple, there was a distinguished and noble priest, named Jōshin. He liked "imo-gashira," meaning taro (one of the root vegetables cultivated in Japan since ancient times), and ate it in large quantities. Even during Buddhist conferences, he would place a large bowl of taro next to him and eat it while reading sutras (canonical scriptures in Buddhism). 

 When he felt ill, he would seclude himself to his room for a week or two for treatment. He personally selected the best taro, consumed large quantities of it, and believed that it would cure his illness. He never allowed anyone else to eat his taro, consuming it entirely on his own. 

**Figure 1:**
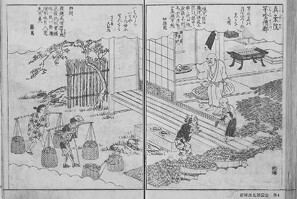
A painting depicting Jōshin. This picture is from "Miyako Rinse Meisho-zue," a tourist brochure from the Edo period (late 18th century, 400 years after Jōshin) for a famous garden in Kyoto. In the section on the Shinjō-in Temple, the episode of Jōshin introduced in this article is illustrated. Jōshin stands in the house on the right-hand side of the picture. Source: This picture is provided as open data under the CC (Creative Commons) license "CC0 (CC0 1.0 worldwide public domain offering)" from the National Archives of Japan Digital Archive (www.digital.archives.go.jp).

 He was very poor during his youth years. Before his master died, he had inherited 200 *kan* and the temple. However, he chose to sell the temple for 100 *kan* and used the total of 300 *kan* (approximately $200,000 today) to purchase taro. He deposited this money with an acquaintance at the center of Kyoto, bought 10 *kan* worth of taro at a time, and consumed it in large quantities. He had no other use for money and spent it entirely on taro (Figure 1). Common people remarked, "He was poor, yet he spent 300 *kan*, a substantial sum, solely on taro. He is a very rare person with a remarkable sense of religious morality." 

 This priest had given another priest the nickname "Shiro-ururi." A person asked him, "What is Shiro-ururi?" To which he replied, "I do not know anything like that either. If there was such a thing, it would look like this priest’s face." 

 The priest was good-looking, robustly built, and had a strong appetite. He excelled in calligraphy, knowledge, and rhetoric, making him a prominent figure in his Buddhist sect. Consequently, he was also regarded as an important priest at Shinjō-in Temple. 

 However, he was an eccentric individual who took the real world for granted. He acted freely on all matters and rarely conformed to the expectations of others. Even at Buddhist meetings and dinners, if food was served to him before it was served to all other attendees, he would immediately eat it himself. When he wished to leave, he would simply stand up and leave alone. 

 Even during regular meals and snacks, he did not eat at a fixed time, similar to the other staff members. If he felt hungry, he would eat at any time, whether in the middle of the night or at dawn. Conversely, if he felt sleepy, he would lock himself in his room during the day and ignore others regardless of their importance. On the other hand, when he was awake, he walked around without sleeping for several days, chanting continuously without distraction. 

 As mentioned above, despite his unusual behavior, he was not disliked by others and was treated with tolerance. This tolerance was likely due to his outstanding virtues and character. 

## DISCUSSION

 According to Kenkō’s description in this essay, the priest, Jōshin exhibited several unusual behaviors. Based on these behaviors, we applied diagnostic criteria for possible bvFTD^5^. 

### Symptom A. Early behavioral disinhibition

 Jōshin ate taro (one of the root vegetables that has been cultivated in Japan since ancient times) during a discussion meeting, ate and slept whenever he wanted, gave others the nickname "Shiro-ururi" and was the only one to eat before others at dinner parties and then leave. 

### Symptom C. Early loss of sympathy or empathy

 Jōshin did not share taro or listen to others, even when they had important matters to discuss. 

### Symptom D. Early perseverative, stereotyped, or compulsive/ritualistic behavior

 Jōshin ate nothing but taro and walked around chanting for several days without sleeping. 

### Symptom E. Hyperorality and dietary changes

 Jōshin was both a big and picky eater, spending his wealth on taro. 

 These episodes correspond to symptoms A, C, D, and E in the diagnostic criteria for bvFTD^5^. Understandably, since imaging tests (III-C) were not performed, the diagnosis remains in the "possible" category according to the criteria. Conversely, since Jōshin was able to perform the duties of a priest, it is suggested that his intelligence, verbal fluency, and executive functions were preserved. Therefore, it is unlikely that he suffered from symptoms B: "early apathy or inertia" or F: "executive/generation deficits with relative sparing of memory and visuospatial functions," or even progressive aphasia, including semantic aphasia and apraxia of speech. Furthermore, and importantly for the diagnosis of bvFTD, Kenkō’s description did not allow us to determine whether Jōshin’s subsequent clinical course was progressive. 

 Jōshin appeared only once in this essay. Another similar anecdote concerns a woman who ate only chestnuts (Chapter 40), which Kenkō did not elaborate on^3,4^. To the best of our knowledge, a document titled "Go-udain Gokanjoki" (Report on the Buddhist empowerment ceremony for the retired emperor Gouda) supports his existence. However, this document does not contain any information regarding his words or actions. Therefore, the age at which his behavioral abnormalities were discovered and his clinical course remain unknown. Based solely on this essay, we can speculate on Jōshin’s age at the time he exhibited the above-mentioned abnormal behavioral abnormalities and his life history up to that point. First, Ninna-ji was a "monzeki" temple whose head priest was a member of the Imperial family. Since Jōshin was entrusted with the duties of a priest at such a prestigious temple and was also given a sub-temple of it, it was suggested that he was middle-aged or older and had not previously exhibited any behavioral abnormalities that would have affected his career advancement. Furthermore, as Kenkō’s described, Jōshin had acquired a wide range of education; therefore, it was assumed that Jōshin had no problems with his learning ability, at least when he was young. 

 We interpreted Jōshin’s act of nicknaming another priest "Shiro-ururi" as indicative of early behavioral disinhibition, although this interpretation may be an overstatement. However, this episode could also be interpreted as a language disorder. The term "Shiro-ururi" is interpreted as a compound of "shiro," which means white, and "ururi," which is an onomatopoeia for rough people^4^. Therefore, this word may represent monemic paraphasia, a type of non-aphasic misnaming^6^. Non-aphasic misnaming has also been reported in patients with frontal and temporal lobe disorders. However, it is challenging to interpret this description solely as monemic paraphasia or indicative of progressive aphasia associated with FTD, including the stereotypy of speech and semantic aphasia. 

 Next, we discuss whether Jōshin’s behavioral abnormalities can be attributed to causes other than bvFTD. First, we consider whether his behavioral abnormalities can be interpreted as falling within the realm of Buddhist customs. In fact, Buddhism is known for its "kairitsu" (strict rules to follow at all times) that restrict diet and sleep, as well as "kugyō" (mortification, ascetic practices). For example, there is the practice of eating only nuts and grass, known as "Mokujiki-gyō." Furthermore, the "Sen-nichi Kaihō-gyō" (1,000-day mountain training) practice includes a "Dō-iri" (entering the hall), a practice of chanting sutras without sleeping or resting for 9 days. However, the fact that Kenkō, who was himself a priest, described Jōshin’s behavioral abnormalities in this essay as extraordinary, we suggest that it was beyond the realm of normality. 

 Second, we consider whether Jōshin’s behavioral abnormalities can be explained by a disorder other than bvFTD. Even today, it remains difficult to distinguish bvFTD from primary psychiatric disorders. It has been noted that half of bvFTD cases are diagnosed as primary psychiatric disorders early on, and that a definitive diagnosis takes time^7^. It has been noted that distinguishing between bvFTD and primary psychiatric disorders requires not only clinical symptoms but also neuroimaging and biomarker evaluation, including genetic screening^7^. In their review, Ducharme and colleagues listed the primary psychiatric disorders that must be differentiated from bvFTD as major depression, bipolar disorder, obsessive-compulsive disorder, schizophrenia, catatonia, neurodevelopmental disorders, and personality traits and disorders, and explained the distinguishing features of clinical symptoms from each^8^. 

 Some of these primary psychiatric disorders that require differentiation from bvFTD can cause symptoms of bvFTD, including the behavioral disinhibition, loss of sympathy or empathy, perseverative, stereotyped, or compulsive/ritualistic behavior, and hyperorality and dietary changes observed in Jōshin. Therefore, we will discuss whether these disorders listed in Ducharme and colleagues’s behavioral abnormalities can be explained by a disord review matched his clinical features. 

 Bipolar disorder, like bvFTD, is accompanied by hyperorality and behavioral disinhibition. What distinguishes it from bvFTD is that disinhibition is accompanied by a sense of grandiosity and invulnerability. However, Kenkō did not mention this in Jōshin. 

 Patients with obsessive-compulsive disorder, like those with bvFTD, exhibit stereotyped and compulsive/ritualistic behaviors. Taro hoarding observed in Jōshin may also be explained as a symptom of obsessive-compulsive disorder. Most patients with obsessive-compulsive disorder have anxiety and an insight that their compulsions are excessive and do not logically prevent dreaded consequences. Kenkō did not mention whether Jōshin had such an anxiety. Some neurodevelopmental disorders, including autism spectrum disorders, are associated with communication and interpersonal interactions, similar to bvFTD. The distinguishing feature between bvFTD and these neurodevelopmental disorders is the progression of symptoms, particularly whether the symptoms are present from childhood. Kenkō’s description did not reveal any early life symptoms of Jōshin. However, given his high status as a priest, it was suggested that he might not exhibit significant behavioral abnormalities in his youth. Patients with pathological personality traits or personality disorders exhibit behavioral disinhibition similar to those with bvFTD. The distinguishing feature is that personality disorders exhibit symptoms of maladaptive traits from early adulthood, accompanied by fear of abandonment, a high degree of self-concerns, self-harm, and an unstable sense of self. Kenkō’s description makes it difficult to confirm any psychiatric symptoms or introspections in Jōshin’s youth. 

 The possibility that Jōshin’s behavioral abnormalities could be attributed to a primary psychiatric disorder rather than to bvFTD cannot be ruled out. However, due to a lack of information about his medical history and insight into his illness, it was difficult to determine whether he had bvFTD or a primary psychiatric disorder. 

 Finally, the question arises as to why Jōshin was able to continue his work as a priest despite the described behavioral abnormalities, even if not due to bvFTD, without disrupting his own or others’ lives. If his behavioral disorder did not interfere with his life, there are two possible explanations. First, it could be attributed to the accepting attitudes of those around him. In particular, it is possible that his noble status made others reluctant to criticize his behavior. Alternatively, Japanese society at the time may have been more tolerant of unusual individuals than contemporary standards. Additionally, many of Jōshin’s duties as priests, such as prayer and sutra reading (canonical scriptures in Buddhism), were routine and may not have required significant interpersonal communication. Consequently, his work may have been less affected by his behavioral abnormalities. 

 Another explanation is that Jōshin’s substantial knowledge before disease onset, serving as a "cognitive reserve," may have helped maintain his quality of life. The "Nun Study" highlighted instances where there was a dissociation between clinical symptoms and pathological findings^9^. Furthermore, higher education levels have been reported to reduce the severity and progression of dementia^10^. Kenkō also speculates that Jōshin’s virtuous character may have contributed to his acceptance and tolerance of others^3,4^. His cognitive reserve likely enabled him to perform his duties effectively as a priest, and despite his unusual behavior, he may not have caused significant trouble to others. In addition, because Jōshin had a quasi-holy nature, it is possible that his behavioral abnormalities were subject to allegorical alterations and interpretations. 

 It is challenging to conclusively determine whether Jōshin had a bvFTD. It is also possible that he may have had another primary psychiatric disease, developmental disorders as mentioned above, or a head injury involving the frontotemporal lobe, rather than dementia. Nonetheless, it is noteworthy that individuals in medieval Japan with bvFTD or similar symptoms were observed and that Jōshin’s unusual characteristics were deemed significant enough to be recorded. 

 In conclusion, we suggested that the present priest, Jōshin, might be diagnosed with bvFTD according to the description of his unique behavioral characteristics written in Tsurezuregusa. Although he exhibited behavioral abnormalities reminiscent of the bvFTD phenotype, we believe that these were insufficient to provide clear evidence of this neurodegenerative disorder. We believe that further historical evidence must be discovered and interpreted to determine whether his behavioral abnormalities were due to bvFTD or another primary psychiatric disorder. Additionally, his successful adaptation was likely due to the tolerance of people around him and/or the education he had acquired before the onset of the disease, which may have functioned as a "cognitive reserve." 

## Data Availability

No new data were generated or analyzed in this study.
